# Feasibility, safety and efficacy of Woven EndoBridge embolization of intracranial aneurysms with the 2 mm height variants

**DOI:** 10.1007/s00234-025-03806-8

**Published:** 2025-11-01

**Authors:** Lukas Goertz, Sophia Hohenstatt, Eberhard Siebert, Hanna Styczen, Alexander Ranft, David Zopfs, Daniel Kaiser, Pawel Krukowski, Marc Schlamann, Jonathan Kottlors, Jan Paul Janssen, Cornelius Deuschl, Franziska Dorn, Markus A. Möhlenbruch, Thomas Liebig, Christoph Kabbasch

**Affiliations:** 1 Faculty of Medicine and University Hospital, Department of Radiology and Neuroradiology, University of Cologne, Cologne, Germany; 2 Department of Neuroradiology, University Hospital Heidelberg, Heidelberg, Germany; 3 Department of Neuroradiology, University Hospital of Berlin (Charité), Berlin, Germany; 4 Institute for Diagnostic and Interventional Radiology and Neuroradiology, University Hospital Essen, Essen, Germany; 5 Department of Radiology and Neuroradiology, Klinikum Hochsauerland, Arnsberg, Germany; 6 Department of Neuroradiology, University Hospital Dresden, Dresden, Germany; 7 Department of Neuroradiology, University Hospital Bonn, Bonn, Germany; 8 Department of Neuroradiology, University Hospital Munich (LMU), Munich, Germany

**Keywords:** Endovascular, Flow-disruption, Intracranial aneurysm, Intrasaccular

## Abstract

**Purpose:**

Small, shallow aneurysms (SSAs) present technical challenges for endovascular treatment with the risk of aneurysm perforation and coil protrusion. The Woven EndoBridge (WEB) device, particularly the lowest height of 2 mm variant, offers a potential alternative option for these aneurysms. This multicenter study evaluates the feasibility, safety and efficacy of the WEB for SSAs ≤ 4.1 mm width.

**Methods:**

A total of 103 aneurysms (mean size: 3.4 ± 1.1 mm, 67 [65%] bifurcation) treated with WEB single-layer sizes ranging from 3 × 2 mm to 4.5 × 2 mm were retrospectively analyzed. Data on procedural success, complications, and angiographic outcomes were collected. The incidence and potential strategies to counteract WEB protrusion were evaluated.

**Results:**

WEB deployment was successful in 97 (94%) cases, but WEB protrusion occurred in 12 (12%) cases. Strategies to counteract WEB protrusion were change to another endovascular technique (*n* = 4), additional stent implantation (n = 4), change to a smaller WEB size (*n* = 3), and repositioning of the WEB (*n* = 1). Procedural thromboembolic complications occurred in 4 (4%) cases, of which 3 were due to protrusion of the WEB, but all were asymptomatic. Two hemorrhagic complications (2%), caused by aneurysm perforation with the microwire in the first case and with the folded WEB tip in the second, resulted in procedural morbidity (2%). Complete and adequate occlusion rates were 82% (42/51) and 90% (46/51) at 6 months and 76% (19/25) and 88% (22/25) at 12 months, respectively.

**Conclusion:**

The 2 mm height WEB demonstrated feasibility and efficacy in the treatment of SSAs with good safety results, making it a viable alternative endovascular option for SSAs.

**Supplementary Information:**

The online version contains supplementary material available at 10.1007/s00234-025-03806-8.

## Introduction

Advances in neurovascular technology have made a wide range of intracranial aneurysm subsets amenable to endovascular treatment [1]. However, there are still aneurysm morphologies that are difficult to treat endovascularly. One such subset is small, shallow aneurysms (SSAs), particularly at bifurcation sites [2]. A small, flat shape is also a common morphological feature of aneurysm remnants after previous treatment, such as coiling or clipping. Conventional coiling is often not possible for these aneurysms because the coils would protrude into the parent artery due to a wide neck [2]. Stent-assisted coiling and flow diversion are usually feasible for sidewall aneurysms, but these methods are difficult to use at bifurcation sites because of the risk of branch or perforator occlusion [3]. In addition, these methods carry an increased risk of thromboembolic events and hemorrhagic complications due to the need for antiplatelet therapy [4]. 

Intrasaccular flow disruption, specifically the Woven EndoBridge (WEB; Microvention, Aliso Viejo, CA, USA), has become an established treatment option for a wide range of aneurysms, particularly wide-necked bifurcation aneurysms [5, 6]. Miniaturization of this device and its deployment system has resulted in the WEB 17 system, which is compatible with a 0.017” microcatheter [7, 8]. In this context, the WEB has become available in sizes as small as 3 × 2 mm (width x height) [6]. In particular, the flat devices with a height of 2 mm are a promising option for SSAs, as they resemble their shallow shape and may allow treatment of wide-necked bifurcation SSAs without stent support.

This retrospective, multicenter study evaluates the safety and efficacy of treating SSAs (≤ 4.1 mm) with the WEB using 2 mm device heights. Technical nuances of this modality are described in detail and the feasibility of this approach is critically evaluated.

## Methods

This is a retrospective study of consecutive patients treated with WEB sizes 3 × 2 mm, 3.5 × 2 mm, 4 × 2 mm, and 4.5 × 2 mm at 7 institutions between September 2018 and April 2024. The study was evaluated by the first author’s Institutional Review Board.

### Inclusion criteria

Inclusion criteria were: (1) successful or failed treatment with the WEB 17 in the above sizes, (2) additional devices implanted (e.g. coils or stent) if performed as a salvage procedure, (3) unruptured/naive, ruptured and recanalized/previously treated aneurysms with a dome width ≤ 4.1 mm and any aneurysm height. The maximum average aneurysm width of 4.1 mm was chosen because the WEB SL 4.5 × 2 mm is suitable for up to this width diameter according to the manufacturer’s sizing chart. Potential exclusion criteria were: (1) mycotic, fusiform, or arteriovenous malformation-associated aneurysms, (2) multimodal approaches with planned WEB implantation in combination with an adjunctive coil and/or stent, and (3) cases without available data on WEB type/size.

### WEB treatment

The low-profile WEB 17 single layer (SL) was used in all procedures. Procedures were performed with a Philips (Best, The Netherlands) or Siemens (Erlangen, Germany) biplane angiosuite via a femoral approach consisting of a triaxial system with a 6 F guiding catheter (e.g., Envoy, Cerenovus, Irvine, CA, USA), an appropriate intermediate catheter (e.g., Sofia 5 F, Microvention), and a 0.017” microcatheter (e.g., VIA, Microvention). WEB sizes were selected based on the manufacturer’s sizing chart, which included aneurysm dome width and height measured in working projections of digital subtraction angiography performed on calibrated images.

The treatment goal was to place the WEB centrally within the aneurysm sac, completely covering the aneurysm neck, but without WEB protrusion. If the optimal WEB position could not be achieved on the first attempt, resheathing maneuvers were performed, including capture and repositioning of the WEB device, change to a different WEB size, or stent implantation in case of WEB protrusion. However, additional stenting was considered only as a salvage option to avoid the need for permanent antiplatelet therapy.

### Anti-aggregation therapy

In elective cases, 100 mg of acetylsalicylic acid (ASA) per os was administered 5–7 days before the procedure and continued for 4–6 weeks thereafter. Ruptured aneurysms were typically treated without antiplatelet therapy. In both scenarios, a single bolus of 5000 IU heparin was administered immediately after inguinal puncture, accompanied by a continuous infusion of 1000 IU/hour aliquots throughout the procedure.

In cases of salvage stent implantation, an intravenous infusion of body weight-adjusted tirofiban was initiated prior to stent placement and maintained for 16–24 h. This was followed by sequential loading with clopidogrel 300 mg per os and additional loading with ASA 250 mg in ruptured cases. After the procedure, a regimen of ASA 100 mg and clopidogrel 75 mg was administered for at least 4 months, followed by permanent ASA monotherapy.

### Data collection and angiographic evaluation

Patient demographics, procedural characteristics, and adverse events were collected retrospectively. Procedure reports were reviewed to identify WEB placement failures and procedural complications. Treatment failure was defined as the inability to adequately position the WEB within the aneurysm sac and subsequent conversion to another treatment modality, such as coiling or clipping.

Adverse events included thromboembolic and hemorrhagic complications. For both types, both clinically symptomatic and technical/asymptomatic complications were recorded. Minor stroke was defined as complete resolution of neurological symptoms within 7 days, whereas major stroke was defined as prolonged symptoms. Procedural morbidity was defined as a 1-point increase on the modified Rankin Scale at discharge compared with baseline.

Dome width (D), height (H), and neck width (N) were measured on DSA scans in working projections, which were also used to select the appropriate WEB size. From these measurements, the dome-to-neck ratio (D/N), the aspect ratio (H/N), and the width-to-height ratio (W/H) were calculated. Aneurysms with neck width ≥ 4 mm and/or D/N ratio ≤ 2 were classified as wide-necked. The WEB/dome ratio reflects the ratio of the nominal WEB width to the dome width used in a particular case.

Follow-up angiography (FU) was performed with DSA, magnetic resonance angiography (MRA), or computed tomography angiography (CTA). Occlusion status was determined as complete occlusion, neck remnant, or aneurysm remnant. Complete occlusion and neck remnants were combined as adequate occlusion. FU duration was stratified into 6-month FU (range 4–8 months), 12-month FU (range 9–15 months), and long-term FU (> 15 months).

### Statistical analysis

Categorical variables were expressed as numbers with percentages. Continuous variables were expressed as mean ± standard deviation. Univariate analyses were performed to identify factors associated with WEB protrusion, thromboembolic events and 6-month incomplete occlusion. Covariates were: Ruptured aneurysm status, recurrent status, posterior circulation, bifurcation location, dome width, height, neck width, dome-to-neck ratio, aspect ratio, width-to-height ratio, wide neck, WEB width, WEB/dome ratio, WEB protrusion and additional stent. Univariate analyses were performed using chi-squared test, Fisher exact test, two-tailed Students t-test and Mann-Whitney U-test, as appropriate. All calculations were performed with SPSS software (IBM SPSS Statistics for Windows, version 25.0, Armonk, NY, USA). A p-value < 0.05 was considered statistically significant.

## Results

### Patient and aneurysm characteristics

The study group consisted of 103 consecutive patients with 103 treated aneurysms after excluding one arteriovenous malformation-associated aneurysm. Baseline patient and aneurysm characteristics are shown in Table 1. Twenty-two (21.4%) aneurysms were ruptured and 6 (5.8%) were recanalized after previous treatment. Aneurysms were located in the anterior circulation in 68 (66.0%) cases and in the posterior circulation in 35 (34.0%) cases, with 67 (65.0%) aneurysms being bifurcation aneurysms. The most common aneurysm locations were the anterior communicating artery (Acom) in 26 (25.2%) cases and the tip of the basilar artery (BA) in 14 (13.6%) cases. The mean dome width was 3.3 ± 0.8 mm with a D/N ratio of 1.3 ± 0.3 and an aspect ratio of 1.3 ± 0.5 mm.

**Table 1:** Baseline patient and aneurysm characteristics

Parameter	Value (*N* = 103)
Patient age (years)	59.2 ± 11.2
Female sex	72 (69.9%)
Ruptured aneurysms	22 (21.4%)
Recurrent aneurysms	6 (5.8%)
Aneurysm location	
Anterior circulation	
Anterior communicating artery	26 (25.2%)
Pericallosal artery	12 (11.7%)
Middle cerebral artery bifurcation	11 (10.7%)
M1-segment	3 (2.9%)
Internal carotid artery	
Terminus	4 (3.9%)
Paraophthalmic segment	4 (3.9%)
Posterior communicating segment	8 (7.8%)
Posterior circulation	
Basilar artery tip	14 (13.6%)
Basilar artery trunk	2 (1.9%)
Superior cerebellar artery	13 (12.6%)
Posterior inferior cerebellar artery	6 (5.8%)
Bifurcation location	67 (65.0%)
Aneurysm size	
Dome width (mm)	3.3 ± 0.8
Height (mm)	3.4 ± 1.1
Neck width (mm)	2.7 ± 0.9
Dome-to-neck ratio	1.3 ± 0.3
Aspect ratio	1.3 ± 0.5
Width-to-height ratio	1.0 ± 0.3
Wide neck	100 (97.1%)

Values are presented either as means with standard deviation or as numbers and percentages

### Procedural details

Procedural details are summarized in Table 2. WEB deployment problems were noted in 20 cases (19.4%), whereby WEB implantation failed in 6 (5.8%) cases: In a ruptured 2 mm Acom aneurysm, the aneurysm ruptured again during probing the aneurysm with the microwire, before planned deployment of a WEB 3 × 2 mm and the implantation was aborted. The patient was thereafter treated by microsurgical clipping. In a 2.5 mm aneurysm of the superior cerebellar artery (SUCA), the WEB 3 × 2 mm spontaneously prolapsed into the parent artery after detachment and was retrieved and the aneurysm treated with a flow diverter. In 3 aneurysms (3 mm internal carotid artery [ICA] terminus - WEB 3 × 2 mm, 3 mm pericallosal artery [PA] - WEB 3 × 2 mm, 4.1 mm posterior communicating artery [Pcom] - WEB 4.5 × 2 mm and 4 × 2 mm) the WEBs protruded into the parent arteries after deployment and before detachment, therefore they were removed and the aneurysms were treated with flow diverter (FD) in the first two cases and with stent-assisted coiling (SAC) in the third case. In a 2.5 mm Acom aneurysm, the microcatheter tip could not be placed centrally in the aneurysm sac due to an unfavorable angle and was unstable, therefore the WEB 4 × 2 mm could not be placed properly, the patient was later treated by clipping.

**Table 2:** Procedural specifics and procedural events

Parameter	Value (*N* = 103)
WEB diameter	
3 mm	27 (26.2%)
3.5 mm	23 (22.3%)
4 mm	27 (26.2%)
4.5 mm	26 (25.2%)
WEB/dome ratio	1.2 ± 0.2
Deployment issues	20 (19.4%)
Treatment failure	6 (5.8%)
Change of WEB size	5 (4.9%)
Defect of WEB detachment	3 (2.9%)
WEB repositioning	3 (2.9%)
Intracranial stent support	4 (3.9%)
Thromboembolic complications	
Asymptomatic/technical	4 (3.9%)
Minor or major stroke	0 (0%)
Hemorrhagic complications	
Asymptomatic/technical	0 (0%)
Subarachnoid/parenchymal hemorrhage	2 (2.0%)
Procedural morbidity	2 (2.0%)

Values are presented either as means with standard deviation or as numbers and percentages

In 5 cases, the initially selected WEB was changed to a WEB of a different size: In 2 aneurysms, the WEB was changed to a larger size due to insufficient wall adaptation - from a WEB 3.5 × 2 mm to a WEB 4 × 2 mm in a 3 × 3 mm recurrent middle cerebral artery (MCA) bifurcation aneurysm and from a WEB 4 × 2 mm to a WEB 4.5 × 2 mm in a 3.5 × 3.5 mm Pcom aneurysm. In 3 aneurysms, the WEB was changed to a smaller size due to protrusion into the parent artery of the originally selected WEB - from a WEB 5 × 3 mm to a WEB 4.5 × 2 mm in a 4.0 × 3.1 mm Acom aneurysm, from a WEB 4.5 × 3 mm to a WEB 4.5 × 2 mm in a 4.1 × 3.4 mm PA aneurysm, and from a WEB 3.5 × 2 mm to a WEB 3 × 2 mm in a 3 × 2 mm BA tip aneurysm.

In 3 cases (3 mm MCA bifurcation - WEB 3.5 × 2 mm, 3.5 mm Acom - WEB 4 × 2 mm, 4.1 mm PA - WEB 4.5 × 2 mm) there was a malfunction in the electrothermal detachment of the WEBs after placement, so they were removed and replaced with a WEB of the same size.

**Fig. 1:**
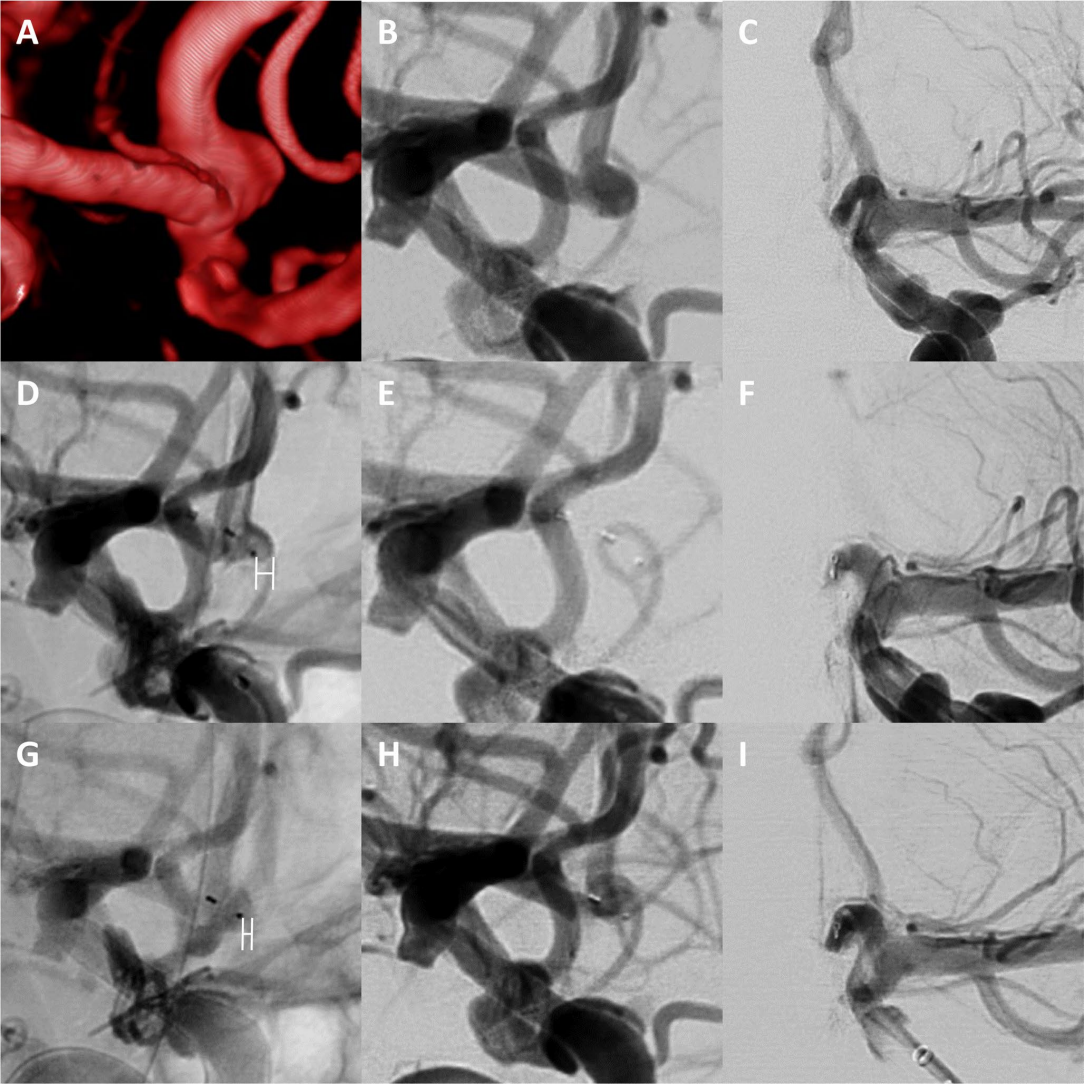
Repositioning of the WEB to counteract WEB protrusion. Three-dimensional reconstructions from rotational angiography (**A**) and digital subtraction angiography in lateral (**B**) and anterioposterior (a.p.) projection (**C**) show an unruptured aneurysm of the anterior communicating artery (width: 3.5 mm, height: 3.2 mm, neck width: 3.3 mm). Note that the patient has previously been treated for two internal carotid artery aneurysms with flow diversion and one middle cerebral artery aneurysm with clipping. Due to the location of the bifurcation, the aneurysm should be treated with a WEB. In a first attempt, a WEB SL 4 × 2 mm was deployed inside the aneurysm (**D**). The pre-detachment control series showed occlusion of the ipsilateral A2 segment, presumably caused by the protrusion of the WEB into the parent artery (**E **+ **F**). In a second attempt, the WEB was placed slightly more distally within the aneurysm sac, as visualised by the distance measurements between the WEB tip and the apex of the aneurysm contour in (**D**) and (**G**). The control series also showed patency of the parent vessels, including the A2 segment (**H **+** I**) and contrast retention within the WEB (not shown). The patient was not yet available for angiographic follow-up

In 3 cases it was necessary to reposition the WEB without changing the WEB size: In the first 2 cases (3 mm BA tip - WEB 3 × 2 mm, 2.3 mm MCA bifurcation - WEB 3 × 2 mm) the WEB was malrotated in the aneurysm sac after the first deployment, which was corrected in the second attempt. In the third case, the WEB 4 × 2 mm protruded into the parent artery of a 3.5 × 3.2 Acom aneurysm, resulting in A2 occlusion due to parent vessel narrowing and potentially apposition thrombus formation. The WEB was then placed slightly more distally into the aneurysm sac, which was successful. The case is presented in Fig. 1.

**Fig. 2:**
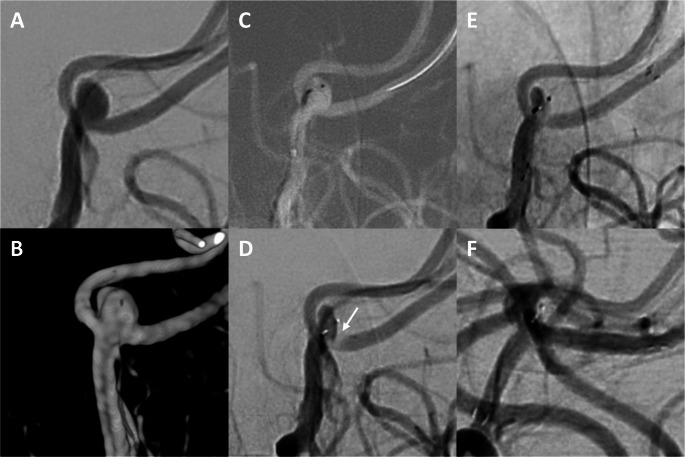
Additional stent implantation to counteract WEB protrusion. Digital subtraction angiography (**A**) and three-dimensional reconstructions from rotational angiography (**B**) show an unruptured aneurysm of the pericallosal artery (width: 3.6 mm, height: 3.0 mm, neck width: 2.1 mm). The aneurysm was probed with a VIA 17 microcatheter and a WEB 17 SL 4 × 2 mm was deployed inside the aneurysm as shown in the roadmap (**C**) and in the DSA scan after detachment (**D**). Due to the slightly irregular shape of the aneurysm, the position of the WEB is also slightly tilted and tends to protrude into the A2 branch, leading to apposition thrombus formation (**D**, arrow). An intravenous infusion of body weight-adjusted tirofiban was initiated, resulting in the dissolution of the thrombus. In addition, a Neurofrom Atlas 3 × 21 mm microstent (Stryker, Kalamazoo, MI, USA) was implanted to prevent thrombus formation (**E**). At the 6-month angiographic follow-up, the aneurysm was completely occluded (**F**) and there was no in-stent stenosis (**F**)

In 4 cases (4 mm MCA bifurcation - WEB 4.5 × 2 mm, 3.5 mm SUCA - WEB 4 × 2 mm, 3.6 mm PA - WEB 4 × 2 mm, 3.1 mm paraophthalmic ICA - WEB 4 × 2 mm) additional stenting was required to counteract WEB protrusion. In the last two cases, a small apposition thrombus formed at the WEB after deployment and was treated with i.v. tirofiban prior to stent implantation. One of these cases is shown in Fig. 2.

In total, WEB protrusion was observed in 12 (11.7%) cases and strategies to address WEB protrusion were removal of the WEB and change to another modality in 4 cases, additional stent placement in 4 cases, change to a smaller WEB size in 3 cases, and repositioning of the same WEB in 1 case. In univariate analysis, larger dome width (3.7 ± 0.9 mm vs. 3.2 ± 0.7 mm, *p* = 0.029) and smaller WEB/dome ratio (1.1 ± 0.1 vs. 1.2 ± 0.2, *p* = 0.014) were associated with WEB protrusion.

### Procedural complications

Procedural thromboembolic complications occurred in 4 cases (3.9%), all due to apposition thrombus formation at the implanted WEB and were medically managed with i.v. tirofiban infusion. All of these events were clinically silent. In the first three cases, the WEB was repositioned or a supportive stent was implanted after thrombus dissolution as described above. In the fourth case, a 2 mm PA aneurysm, the correctly placed 3 × 2 mm WEB was left in place after tirofiban administration and an unremarkable control series at 10 min. In the univariate analysis, WEB protrusion (3/12 [25.0%] vs. 1/91 [1.1%], *p* = 0.005) and additional stent placement (2/4 [50.0%] vs. 2/99 [2.0%], *p* = 0.007) were associated with thromboembolic complications (Supplemental Table).

Hemorrhagic complications occurred in 2 cases of ruptured aneurysms. The first was the ruptured 2 mm Acom aneurysm described above, where the aneurysm re-ruptured during probing the aneurysm with the microwire and WEB deployment was aborted. In the second case, a ruptured 4 mm Acom aneurysm re-ruptured upon deployment and detachment of the WEB 4.5 × 2 mm, and the bleeding was controlled with a two-minute balloon occlusion of the parent artery. In both cases the hemorrhagic complication was considered symptomatic as both patients had morbidity at discharge (case 1: mRS 1, case 2: mRS 4), although the morbidity may have been caused by the initial subarachnoid hemorrhage (SAH).

In summary, there were no minor or major ischemic strokes. There were 2 procedural morbidities caused by aneurysm perforation and 2 deaths in SAH patients that were considered SAH-related and not related to treatment.

### Angiographic results

A total of 67 patients were available for 104 angiographic control scans at different follow-up (FU) periods, as summarized in Table 3. Complete and adequate occlusion rates were 42/51 (82.4%) and 46/51 (90.2%) for 6-month FU, 19/25 (76.0%) and 22/25 (88.0%) for 12-month FU, and 20/28 (71.4%) and 27/28 (96.4%) for long-term FU. There were 35 patients with more than one FU, whereof recanalization beyond the 6-month FU was observed in 3 cases (8.6%). All of these were neck remnants due to a slight WEB compression and did not require retreatment. In contrast, 3 aneurysms with neck or aneurysm remnants on the first angiographic FU were retreated within 12 months after treatment: a neck remnant of a paraophthalmic ICA aneurysm was retreated with SAC, an aneurysm remnant of a PICA aneurysm was retreated with FD implantation, and an aneurysm remnant of a SUCA aneurysm was retreated with SAC. In univariate analysis, sidewall location (7/11 [63.6%] vs. 2/31 [6.4%], *p* = 0.020), larger dome width (3.8 ± 0.7 mm vs. 3.2 ± 0.6 mm, *p* = 0.010), larger height (4.5 ± 2.1 mm vs. 3.1 ± 0.6 mm, *p* < 0.001) and larger neck width (3.4 ± 1.1 mm vs. 3.1 ± 0.6 mm, *p* = 0.021) were associated with incomplete occlusion at 6 months (Supplemental Table).

**Table 3:** Angiographic outcome

	6-month FU (*n* = 51)	12 month FU (*n* = 25)	Long-term FU (*n* = 28)
FU period	5.5 ± 1.7	11.1 ± 2.0	22.3 ± 8.3
Complete occlusion	42 (82.4%)	19 (76.0%)	20 (71.4%)
Neck remnant	4 (7.8%)	3 (12.0%)	7 (25.0%)
Aneurysm remnant	5 (9.8%)	3 (12.0%)	1 (3.6%)
Recanalization to previous FU	-	0/15 (0%)	3/26 (11.5%)
Retreatment	1 (2.0%)	2 (8.0%)	0 (0%)

*FU *follow-up

## Discussion

In the present study, WEB embolization of SSAs was feasible in 94%, with 19% experiencing deployment problems and 4% requiring stent support. The overall complication rate was 6%, 2% of which were symptomatic. The adequate occlusion rate increased from 90% at 6 months to 96% at long-term follow-up, and the recanalization rate was 9%. From a technical and procedural point of view endovascular treatment of SSAs still remains a highly challenging and cumbersome approach. To our knowledge, this is the first study focused on WEB embolization of SSAs using the 2 mm height variants.

There are few clinical studies of the WEB 17 in unselected cohorts of small aneurysms. The technical success rates and the need for additional stents were 98% and 10% in the study by Maurer et al., 95% and 9% in the study by Pagano et al. and 100% and 4% in the prospective CLEVER study (CLinical EValuation of WEB 17 device in intracranial aneuRysms), respectively [7–9]. Hence, the feasibility rates in these studies were slightly better than in the present study (94%), while a similar subset of patients required an additional stent (4%).

However, it should be noted that the present study focused on SSAs characterized by a small aneurysm height (3.4 mm) and a low aspect ratio (1.3). In the previous WEB 17 studies, these parameters were larger, with a height of 4.5 mm in the study by Maurer et al. and a maximum diameter of 5.1 mm in the CLEVER study [7, 9]. 

Due to the flat shape of the aneurysm, WEB protrusion into the parent artery was the most common technical problem, occurring in 12 cases (12%). This rate is higher than in the WEBCAST/WEBCAST-2/French Observatory benchmark studies where protrusion was observed in only 4% of cases [10]. Despite the shallow aneurysm morphology, the principle of WEB oversizing was consistently applied, as represented by a WEB/dome ratio of 1.2. In the univariate analysis, WEB protrusion was associated with larger dome width and smaller WEB/dome ratio. This association seems unlogical and cannot be explained by the authors, as WEB protrusion would be expected with smaller aneurysm heights and more pronounced oversizing (WEB/dome ratio).

Several strategies to address WEB protrusion have been identified in this study. Post-deployment repositioning of the WEB without changing to a different WEB size is straightforward if the WEB was deployed too low in the aneurysm and there is free space in the upper aneurysm dome. Changing to a smaller WEB size was also efficient when the originally selected WEB size proved to be too large, even when selected according to the manufacturer’s sizing chart. In the present study, selecting a smaller size resolved WEB protrusion in 3 cases. An established method to counteract WEB protrusion is additional stent implantation [11]. However, this should only be used as a salvage option, as stenting nullifies the advantages of the WEB as an intrasaccular device because it requires antiplatelet medication and is associated with an increased risk of procedural events [11]. The same applies to a switch to another treatment modality, particularly if the aneurysm requires stenting or clipping.

Overall, 9% of aneurysms could not be treated with WEB only as originally planned, with either stenting or clipping required in these cases. Although the rate of additional stent implantation did not exceed that of other WEB studies, we recommend that SSAs be treated with dual antiplatelet therapy to allow for additional stent implantation or conversion to another stent-assisted modality, if required [10]. 

The clinically relevant issues of WEB protrusion are the risk of apposition thrombus formation at the protruding WEB portion with occlusion or distal embolization and narrowing of the parent artery [6]. In the present study, procedural thromboembolic complications occurred in 4%, of which 3% were due to WEB protrusion, but none were symptomatic. The association between WEB protrusion and thromboembolic complications has been shown in the univariate analysis. Previous WEB 17 studies reported comparable rates of thromboembolic events with 4% (1% symptomatic) in the study by Maurer et al., 7% in the study by Pagano et al., and 6% (2% symptomatic) in the CLEVER study [7–9]. 

For endovascular coiling, treatment of small aneurysms is associated with an increased procedural risk of aneurysm perforation. In this context, the procedural rupture rate was 8% for small aneurysms ≤ 3 mm versus 4% for larger aneurysms in the study by van Rooij et al.[12] Similarly, in WEB procedures, there may be an increased risk of aneurysm perforation with the tip of the folded WEB device due to the shallow anatomy. To avoid this complication, the WEB must be opened very proximal, approximately at the level of the neck, and after partial unfolding, pushed distally into the aneurysm. Iatrogenic aneurysm perforation with the WEB tip occurred in 1 case in the present series. This complication could be managed by leaving the deployed WEB into the aneurysm to provide stasis and additional temporary balloon occlusion of the parent artery. Another hemorrhagic complication occurred during probing the aneurysm with the microwire. For comparison, hemorrhagic events with WEB 17 occurred in 0.8% in the study by Maurer et al., 0.9% in the study by Pagano et al., and 0.6% in the CLEVER study [7–9]. 

After endovascular treatment, small aneurysms usually have better occlusion results than large aneurysms, and large aneurysm size is a risk factor for recanalization. In this context, van Rooij et al. reported lower retreatment rates after coiling of small aneurysms (5%) compared to large aneurysms (10%).[12] A wider neck and larger aneurysm size also correlate with incomplete occlusion after WEB treatment, as larger aneurysms require more time for complete intrasaccular thrombosis to occur [13, 14]. In this context, studies using the WEB 17 for small aneurysms < 7 mm report higher occlusion rates than WEB studies on larger sizes. Complete and adequate occlusion rates were 71% and 93% in the WEB 17 study by Maurer et al. and 68% and 86% in the WEB 17 study by Pagano et al.[8, 9] In the present study of SSA, the 12-month angiographic results showed complete occlusion in 76% and adequate occlusion in 88%, confirming the high efficacy of WEB embolization for small aneurysms. Notably, there were no major recanalizations leading to aneurysm remnants beyond the 6-month follow-up. In contrast, complete and adequate occlusion rates of 54% and 85% were reported in the WEB-IT study, which included aneurysms with a mean diameter of 6.4 mm treated by diverging WEB types [15]. 

For selected aneurysms, other endovascular techniques might a viable approach for small aneurysms. For coiling, van Rooij et al. reported feasibility in 196 aneurysms ≤ 3 mm with a procedural morbidity rate of 2% and a high 6-month adequate occlusion rate of 94%.[12] Gao et al. reported complete and adequate occlusion in 84% and 93% after coiling and 89% and 100% after SAC with a low-profile visualized intraluminal support (LVIS) device (Microvention) and low morbidity in both groups [16]. Zheng et al. reported 93% complete occlusion and 8% complication rate for SAC with various stents [17]. Flow diversion is also a viable option for small sidewall aneurysms, even though there are no specific series for this aneurysm subset.

However, the studies cited seem to focus on sidewall aneurysms. In particular, coiling of bifurcation aneurysms is often not feasible due to a wide neck, and stent-assisted coiling or flow diversion may be complicated by thromboembolic events [3]. In contrast, the proportion of bifurcation aneurysms in the present study was 65% and almost all aneurysms were wide-necked, which represents a challenging morphology for conventional endovascular treatment. Therefore, some of the aneurysms presented in this series might have been treated by clipping if the WEB had not been available. However, the favorable results of the present study support the WEB as a promising endovascular option for SSAs, including wide-necked bifurcation aneurysms.

### Limitations

This study has several limitations. The results are based on a retrospective analysis with a moderate number of patients. Short- and medium-term follow-up is incomplete, and long-term angiographic follow-up information is lacking due to current unavailability. There is no control group, which limits generalizability. Finally, the lack of core laboratory evaluation raises the possibility of confounding the reported angiographic and clinical results.

## Conclusions

Treatment of small shallow aneurysms with the WEB device was feasible in the majority of cases and associated with low procedural morbidity. Technical care must be taken in selecting the appropriate device and positioning the WEB within the aneurysm sac to avoid WEB protrusion and aneurysm perforation with the tip of the folded WEB. This series presents several strategies to counteract WEB protrusion, including device repositioning, changing to a smaller WEB size, and additional stent implantation. The mid-term complete and adequate occlusion rates are satisfactory and appear to be comparable to other series of the WEB 17.

## Supplementary Information

Below is the link to the electronic supplementary material.

Supplementary Material 1 (DOCX 14.3 KB)

## Data Availability

No datasets were generated or analysed during the current study.
