# Supplementary material for: Feasibility, safety and efficacy of Woven EndoBridge embolization of intracranial aneurysms with the 2 mm height variants
Source: Neuroradiology. 2025 Nov 1;67(11):3229–38. doi: 10.1007/s00234-025-03806-8 (PMC12743084; doi:10.1007/s00234-025-03806-8)
Supplement: Supplementary file 1 — Supplementary Material 1 (DOCX 14.3 KB) [file 234_2025_3806_MOESM1_ESM.docx]

| Parameter | **WEB protrusion** (N=12) | Control (N=91) | P-value | **Thromboembolic complication** (N=4) | Control (N=99) | P-value | **Incomplete occlusion** (N=9) | Control (N=42) | P-value |
| --- | --- | --- | --- | --- | --- | --- | --- | --- | --- |
| Ruptured aneurysms | 3 (25.0%) | 19 (20.9%) | 0.716 | 1 (25.0%) | 21 (21.2%) | 1.0 | 1 (11.1%) | 7 (16.7%) | 1.0 |
| Recurrent aneurysms | 0 (0%) | 6 (6.6%) | 1.0 | 0 (0%) | 6 (6.1%) | 1.0 | 1 (11.1%) | 3 (7.1%) | 0.552 |
| Posterior circulation | 3 (25.0%) | 32 (35.2%) | 0.747 | 0 (0%) | 35 (35.4%) | 0.297 | 5 (55.6%) | 16 (38.1%) | 0.460 |
| Bifurcation location | 8 (66.7%) | 59 (64.8%) | 1.0 | 3 (75.0%) | 64 (64.6%) | 1.0 | 2 (22.2%) | 29 (69.0%) | 0.020 |
| Dome width (mm) | 3.7±0.9 | 3.2±0.7 | 0.029 | 3.1±0.7 | 3.3±0.8 | 0.562 | 3.8±0.7 | 3.2±0.6 | 0.010 |
| Height (mm) | 3.5±1.7 | 3.3±1.0 | 0.696 | 3.0±0.3 | 3.4±1.0 | 0.447 | 4.5±2.1 | 3.1±0.6 | <0.001 |
| Neck width (mm) | 3.1±1.0 | 2.7±0.8 | 0.148 | 2.5±0.6 | 2.8±0.9 | 0.471 | 3.4±1.1 | 2.7±0.7 | 0.021 |
| Dome-to-neck ratio | 1.3±0.3 | 1.2±0.3 | 0.928 | 1.3±0.3 | 1.2±0.3 | 0.892 | 1.2±0.2 | 1.3±0.4 | 0.509 |
| Aspect ratio | 1.1±0.3 | 1.3±0.5 | 0.219 | 1.2±0.2 | 1.3±0.5 | 0.785 | 1.5±0.8 | 1.2±0.4 | 0.235 |
| Width-to-height ratio | 1.2±0.3 | 1.0±0.3 | 0.076 | 1.0±0.2 | 1.0±0.3 | 0.994 | 1.0±0.4 | 1.1±0.2 | 0.384 |
| Wide neck | 12 (100%) | 88 (96.7%) | 1.0 | 4 (100%) | 96 (97.0%) | 1.0 | 9 (100%) | 39 (92.9%) | 1.0 |
| WEB width (mm) | 3.8±0.7 | 3.7±0.6 | 0.602 | 3.8±0.5 | 3.8±0.6 | 0.993 | 3.9±0.4 | 3.7±0.6 | 0.209 |
| WEB/dome ratio | 1.1±0.1 | 1.2±0.2 | 0.014 | 1.3±0.2 | 1.2±0.2 | 0.401 | 1.1±0.2 | 1.2±0.2 | 0.072 |
| WEB protrusion | - | - | - | 3 (75.0%) | 9 (9.1%) | 0.005 | 2 (22.2%) | 3 (7.1%) | 0.209 |
| Additional stent | - | - | - | 2 (50.0%) | 2 (2.0%) | 0.007 | 1 (11.1%) | 3 (7.1%) | 0.552 |

Supplemental Table: Univariate analysis of factors potentially associated with WEB protrusion, thromboembolic events and incomplete occlusion at 6 months.
